# A SCOPING REVIEW OF SOCIAL DETERMINANTS OF HEALTH IN CHARLES BONNET SYNDROME RESEARCH

**DOI:** 10.1093/geroni/igad104.3151

**Published:** 2023-12-21

**Authors:** Joe Strong, Takashi Amano, Isabella Hoàng, Hannatu Amaza, Rachel Tilsen

**Affiliations:** University of Wisconsin - Madison, Madison, Wisconsin, United States; Rutgers University, Newark, New Jersey, United States; University of Wisconsin - Madison, Madison, Wisconsin, United States; University of Wisconsin - Madison, Madison, Wisconsin, United States; University of Wisconsin School of Medicine and Public Health, Madison, Wisconsin, United States

## Abstract

Charles Bonnet Syndrome (CBS) is a neurological condition featuring pseudohallucinations that affects previously sighted individuals, many of whom are older adults. The CBS literature primarily concerns prevalence estimates, neurological studies, and experiences of people living with the condition; it is not clear what is known concerning social determinants of health (SDoH). We conducted a scoping review guided by Healthy People 2030 SDoH categories including Economic Stability, Education Access & Quality, Health Care Access & Quality, Neighbourhood & Built Environment, and Social & Community Context. Research articles included in this study represent 5 countries on 5 different continents. Sample size ranged from 5 to 88 across all included studies (M = 40.25, Median = 33). Two studies did not report enough info to extract mean age; Mean of mean age across the remaining 6 studies is 63.25 (Range: 27.8-79.47). Across the 8 included articles, 9 different SDoH variables were identified, representing 4 of the 5 Healthy People 2030 SDoH categories, with the exception of Health Care Access & Quality. All studies reported age and gender, most reported education (n = 6, 75%) and living arrangement (n = 5, 62.5%), and half (n = 4) reported marital status. Few studies, however, reported race (n = 2, 25%), loneliness/social life (n = 2, 25%), occupational status (n = 1, 12.5%), and living environment (n = 1, 12.5%). This study highlights the need for expanded inclusion of SDoH variables in CBS research to fill gaps in the knowledge base in this understudied condition.

